# The role of inflammation in central serous chorioretinopathy: From mechanisms to therapeutic prospects

**DOI:** 10.3389/fphar.2024.1200492

**Published:** 2024-05-21

**Authors:** Xiao Shen, Fanhua Kong, Jing Wen, Xiao Wang, Chunlian Huang

**Affiliations:** ^1^ Department of Ophthalmology, Taizhou Central Hospital ( Taizhou University Hospital), Taizhou, Zhejiang, China; ^2^ Zhongnan Hospital of Wuhan University, Institute of Hepatobiliary Diseases of Wuhan University, Transplant Center of Wuhan University, National Quality Control Center for Donated Organ Procurement, Hubei Key Laboratory of Medical Technology on Transplantation, Hubei Clinical Research Center for Natural Polymer Biological Liver, Hubei Engineering Center of Natural Polymer-Based Medical Materials, Wuhan, China

**Keywords:** central serous chorioretinopathy, inflammation, cytokines, chemokines, therapy

## Abstract

Central serous chorioretinopathy (CSC) is a leading cause of permanent vision loss, ranking fourth among macular diseases, trailing only age-related macular degeneration, diabetic retinopathy, and retinal vein obstruction. While mounting evidence implicates inflammation as a pivotal factor in the onset and advancement of CSC, the specific pathophysiological process and molecular mechanisms underlying inflammation remain incompletely understood. A complex network of cytokines, chemokines, and adhesion molecules interplay to trigger inflammatory and pathological cascades, highlighting the need for a comprehensive comprehension of the inflammation-related mechanisms behind CSC progression. In this piece, we examine the existing comprehension of CSC’s pathology and pathogenesis. Additionally, we present an overview of the mechanisms underlying the onset and progression of CSC inflammation, followed by a thorough analysis and discussion of the potential of targeted inflammatory intervention for both preventing and treating CSC.

## 1 Introduction

Central serous chorioretinopathy (CSC) ranks as the fourth most prevalent macular disease, trailing only age-related macular degeneration, diabetic retinopathy, and retinal vein obstruction. This condition can result in irreversible vision loss, making it a significant concern in the field of ophthalmology (Pauleikhoff et al., 2021). Serous retinal detachment (SRD) is a hallmark of CSC, frequently affecting the macular region and accompanied by focal pigment epithelial detachment (PED). These factors can lead to severe visual impairment, with chronic visual impairment being a particularly concerning outcome. As a result, clinicians worldwide have directed their focus toward identifying potential risk factors associated with CSC development (Gawęcki et al., 2019; Mrejen et al., 2019; Gawęcki et al., 2020). Currently, the complete etiology of CSC remains elusive, with the leading factors being thought to be irregularities in the choroid and malfunctions in the retinal pigment epithelium (RPE) (Daruich et al., 2015). Nonetheless, CSC is believed to be a complex ailment that is intricately tied to choriodynia or vascular fever (Berger et al., 2021). As per reports, CSC has a prevalence of 9.9 cases per 100,000 men and 1.7 cases per 100,000 women (Kitzmann et al., 2008). Research indicates that CSC is associated with several recognized risk factors, including but not limited to type A personality, psychosocial stress, corticosteroid use, endogenous hypercorticosis, obstructive sleep apnea, *Helicobacter pylori* infection, phosphodiesterase-5 inhibitors (sildenafil, tadalafil), elevated cortisol levels, and pregnancy (Spiers et al., 2014; Yavaş et al., 2014; Nicholson et al., 2018).

The present understanding of the pathophysiology of CSC is incomplete, particularly with regard to the influence of inflammation on the disease’s development. Multiple possible mechanisms at the upstream, molecular, and pathological levels may play a part in choriogenic permeability changes that lead to CSC. These factors may act in conjunction to produce varying levels of severity in individuals with CSC. The presence of pro-inflammatory cytokines has been linked to CSC, while stimulation of mineral corticosteroid receptors can cause choroidal vasodilation, resulting in the thickening of the choroid and further advancement of CSC.

Despite thorough investigations, the underlying pathophysiology of CSC remains to be comprehensively explained. It is believed that choroidal congestion, heightened permeability, and dysfunction of the retinal pigment epithelium contribute to the development of CSC (Zarnegar et al., 2023). The current focus of research centers on the role of the choroid in the development of CSC. The choroid, a vascular layer located beneath the retina, is responsible for supplying oxygen and nutrients to the outer and middle layers of the retina. It is believed that dysfunction of the choroid is the primary factor contributing to the onset of CSC (Cheung et al., 2019). In 1967, Gass was the first to suggest that CSC arises from hyperpermeability of the choroid, which leads to an increase in hydrostatic pressure within the choroid tissue (Gass, 1967). Despite other theories being investigated, Gass’s proposal has been further supported by imaging studies employing indocyanine green angiography (Gass, 1967).

In the end, numerous molecular and physiological elements play a role in the development of choroidal hypertonicity and consequent serous retinal detachment. These pathophysiological factors encompass a broad range, including vortex vein congestion or compression, activation of mineral corticosteroid receptors, dysregulation of the complement pathway, inflammatory processes, and oxidative stress. The precise impact of inflammation on the choroid in the pathogenesis of CSC remains unclear, particularly in light of the connection between the development of CSC and exposure to either endogenous or exogenous steroids. While steroids have been shown to exacerbate rather than alleviate CSC, the proposed inflammatory mechanism responsible for the condition may be characterized as steroid-insensitive inflammation (Tittl et al., 1999). Despite the identification of heightened levels of pro-inflammatory cytokines, such as IL-6, in certain individuals with CSC, a definitive association between systemic inflammation and CSC has yet to be firmly established (Jung et al., 2014).

Our analysis centers on the contribution of inflammation to the pathophysiology of CSC, along with a comprehensive overview of the latest breakthroughs in both established and experimental treatments for CSC.

## 2 Classification of CSC

**FIGURE 1:**
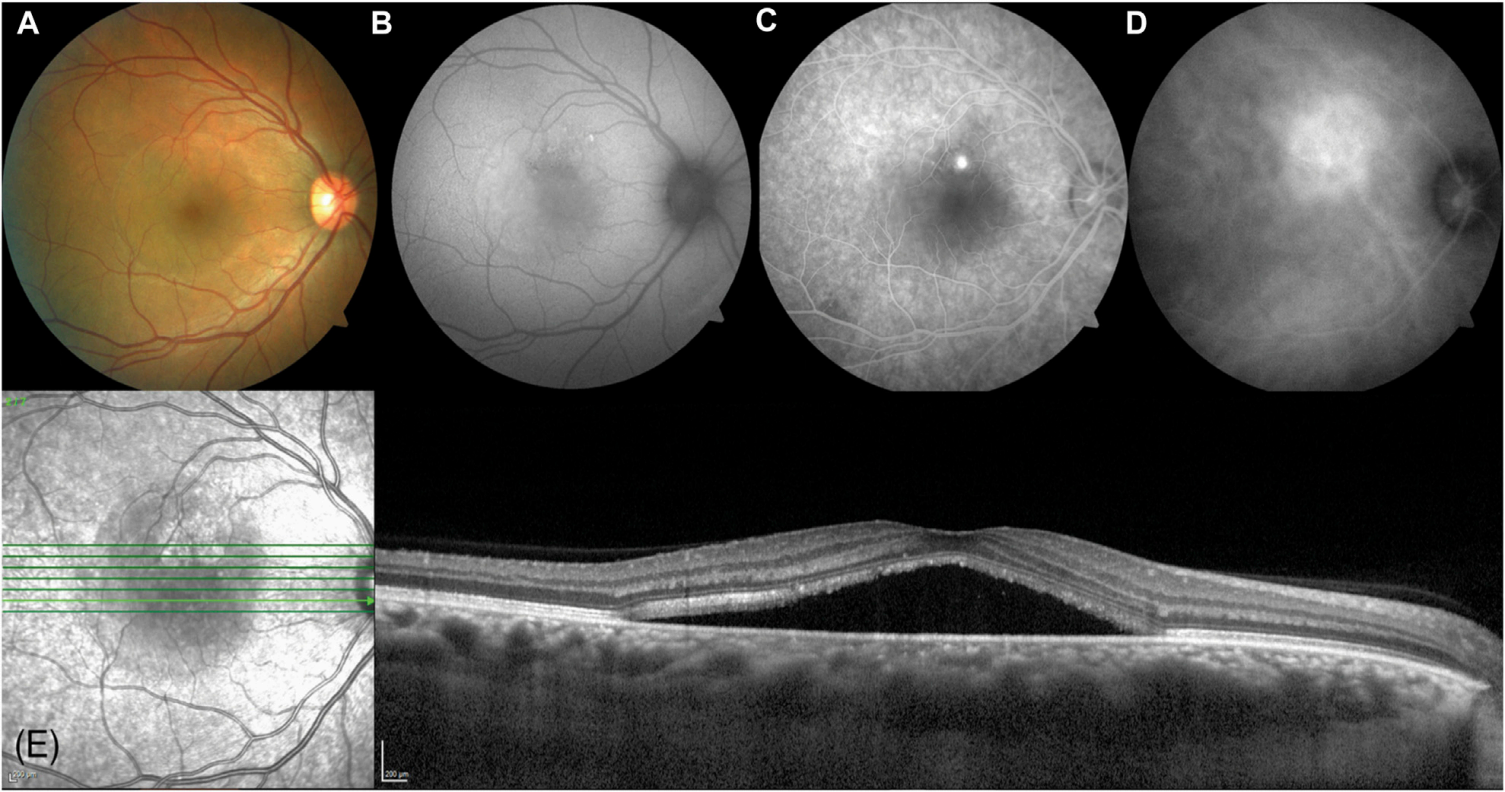
A 38-year-old Arabic male presented with a 2-month history of reduced vision in his right eye (visual acuity right 6/9.5, left 6/7.5). **(A)** A circular blister of subfoveal fluid is seen at the macula (VISUCAM^®^ 500, Carl Zeiss Meditec AG). **(B)** A circle of autofluorescent changes is seen in the area of subretinal fluid. This is hyperautofluorescent on the temporal side, and hypoautofluorescent on the nasal side. **(C)** Fundus fluorescein angiography shows a single spot of hyperfluorescent leakage (“inkspot”) just superior to the fovea. **(D)** Indocyanine green angiography shows a larger area of hyperfluorescence superonasal to the fovea. **(E)** Enhanced depth imaging optical coherence tomography (SPECTRALIS^®^ HRA + OCT, Heidelberg Engineering Inc.) shows a subfoveal fluid with a thickened choroid (553 microns). Reprinted with permission from Fung et al. (2023). Central serous chorioretinopathy: A review. Clin Exp Ophthalmol. 10.1111/ceo.14201.

CSC may be categorized into acute, chronic, and recurrent subtypes, which are determined by various factors, such as the length of time that subretinal fluid persists, whether the fluid has receded in between episodes, and the observable indications in the fundus. (Fung et al., 2023). The majority of CSC cases are acute (Figure 1) (Fung et al., 2023), with a favorable prognosis. Typically, acute CSC is self-limiting and resolves spontaneously within 3–4 months. During this timeframe, most patients experience reabsorption of subretinal fluid and an improvement in visual function (Gilbert et al., 1984; Loo et al., 2002). Blurred, distorted, and diminished vision is commonly reported by individuals experiencing acute CSC; however, significant vision loss seldom occurs following remission (Daruich et al., 2017a). Along with the observable subretinal detachment syndrome (SRDS) during fundus examination, spectral domain optical coherence tomography (SD-OCT) can reveal specific changes in the retinal pigment epithelium (RPE), such as pigment epithelial detachments (PEDs). Additionally, fluorescein angiography (FA) frequently detects RPE leakage. Typically, subretinal fluid (SRF) resolves within 3–4 months without any long-term consequences. For instance, a study involving 27 individuals with CSC who were monitored for an average of 23 months demonstrated that all patients experienced spontaneous resolution of SRF within approximately 3 months. Furthermore, all eyes returned to optimal corrected vision, and all but two patients regained 6/9 or better visual acuity (Klein et al., 1974). On the other hand, chronic or recurrent CSC can result in significant visual impairment due to retinal pigment epithelium (RPE) alterations and sensory nerve layer atrophy (Baran et al., 2005). Hence, it is vital to monitor and manage risk factors as a suitable intervention for individuals with acute CSC (Wang et al., 2008).

**FIGURE 2:**
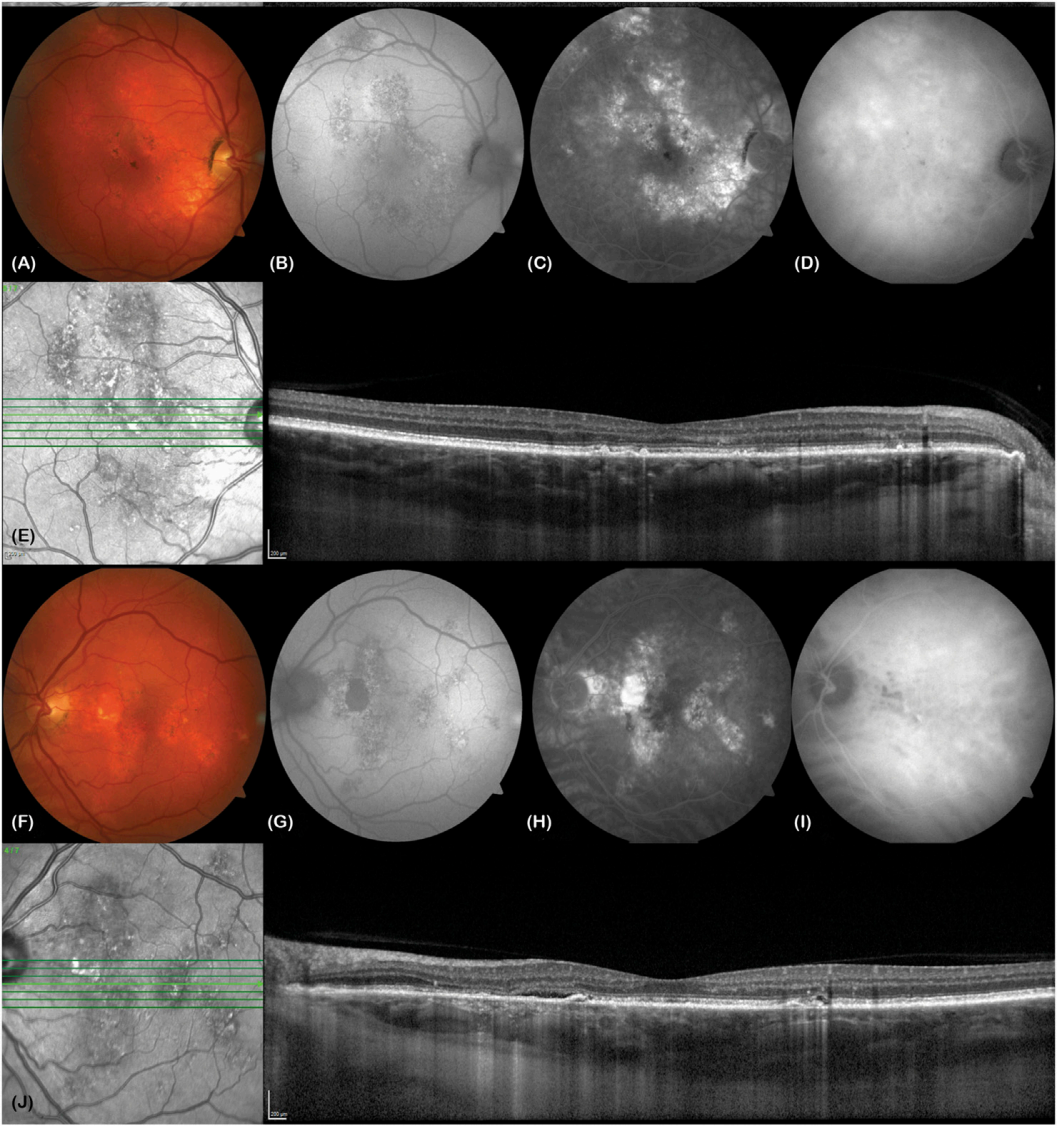
A 63-year-old Caucasian male truck driver presented with poor vision in his left eye over 20  years (visual acuity right eye 6/6, left eye 6/6). He described metamorphopsia, poor stereopsis and monocular diplopia. **(A and F)** Colour fundus photos show bilateral retinal pigment epithelial changes in both eyes (VISUCAM^®^ 500, Carl Zeiss Meditec AG). **(B and G)** These correspond to areas of stipple autofluorescence. There is a patch of hypoautofluorescence nasal to the left macula due to atrophy. **(C and H)** Fundus fluorescein angiography shows bilateral hyperfluorescent window defects in both eyes. **(D and I)** Mid-phase indocyanine green angiography shows bilateral hypercyanesence due to choroidal hyperpermeability. **(E)** Enhanced depth imaging optical coherence tomography (EDI-OCT, SPECTRALIS^®^ HRA + OCT, Heidelberg Engineering Inc.) of the right macula shows some pachydrusen, irregularity of the ellipsoid zone and a thickened choroid (subfoveal choroidal thickness of 503 microns) with pachyvessels in Haller’s layer. **(J)** EDI-OCT of the left macula shows a slick of subretinal fluid and ellipsoid zone loss nasal to the fovea, tiny pigment epithelial detachments nasal and temporal to the fovea, a thickened subfoveal choroidal thickness of 501 microns and pachyvessels. Reprinted with permission from Fung et al. (2023). Central serous chorioretinopathy: A review. Clin Exp Ophthalmol. 10.1111/ceo.14201.

Figure 2 (Fung et al., 2023) illustrates the distinguishing features of Chronic Central Serous Chorioretinopathy (CSC). This particular type of CSC is marked by the continuous presence of subretinal fluid, lasting anywhere from 4 to 6 months. This condition is observed in a minority of CSC cases, constituting around 5%–15% of diagnoses, and is usually detected through the use of optical coherence tomography (OCT). (Yannuzzi, 2010; van Rijssen et al., 2019). One of the key hallmarks of this condition is the appearance of extensive areas of RPE atrophy, often accompanied by a decrease in fundus autofluorescence (Teke et al., 2014). Unfortunately, in cases of chronic CSC, the RPE and photoreceptor cells can become permanently damaged and lead to irreversible visual impairment due to progressive damage to the retina (Daruich et al., 2015; You et al., 2023). Several factors increase the likelihood of developing chronic CSC, including having a subfoveal choroid thickness of ≥500 μm, an elevated PED of ≥50 μm at the leakage site, being aged ≥40 years, and having increased subretinal matter (Daruich et al., 2017b). It is worth noting that acute CSC can also lead to recurrent episodes of subretinal fluid and persistent SRF, which can lead to chronic CSC (Levine et al., 1989). Moreover, 1 year from the initial manifestation of CSC, a substantial portion of patients, ranging from 30% to 50%, will undergo a recurrence of SRF, which will subsequently resolve without any intervention. Although the medical community has yet to reach a unanimous consensus on the specific parameters that define chronic CSC, a majority of experts currently define it as an ongoing accumulation of fluid that persists for a minimum of 3–6 months (Negi and Marmor, 1984). At present, photodynamic therapy (PDT) has demonstrated promising results in mitigating choroidal hyperosmosis and chronic CSCS-associated RPE leakage. Notably, PDT has also exhibited its capacity to safeguard both anatomic functionality and vision in individuals afflicted with CSC (Taban et al., 2004). Chronic CSC is often linked to a less favorable visual prognosis over the long term when compared to acute CSC. This is due in part to several contributory factors, such as cystic macular degeneration, choroidal neovascularization (CNV), outer retinal membrane damage, advanced age at initial diagnosis, and poor visual acuity at the onset of symptoms (Breukink et al., 2017; Mrejen et al., 2019).

Recurrence of CSC may manifest in certain patients, even if the subretinal fluid had previously resolved during the early stages of the illness. This recurrence is commonly referred to as recurrent CSC (Singh et al., 2019; Chhablani and Cohen, 2020). Throughout a 22-year follow-up period, around 31% of individuals with CSC encountered relapse, with a median relapse interval of 1.3 years (range 0.4–18.2 years) and an average of 1.5 relapses per year (range 1–2 years) (Kitzmann et al., 2008). Several studies examining long-term outcomes have reported recurrence rates ranging from 30% to 50% among CSC patients (Matet et al., 2018; van Rijssen et al., 2019; Venkatesh et al., 2020). Standardized treatment protocols can help mitigate the likelihood of CSC recurrence. As the such, timely and expedient intervention is paramount in reducing the incidence of CSC relapse.

The acute or chronic form of CSC is a continuous process, but the different pathophysiology and the different disease spectrum throughout the disease course remain unclear. In addition, patients with sensory dissociation of the external foveal nerve at the time of onset may not experience any visual symptoms until the fovea is involved later in the course of the disease and may be misclassified as part of an acute CSCR based on symptom duration. Despite the existence of different nomenclature for CSCS, inconsistencies in the disease persist even after the classification of the disease into acute, chronic, persistent, relapsing, or inactive subtypes (Daruich et al., 2015).

## 3 Inflammation in CSC

**FIGURE 3:**
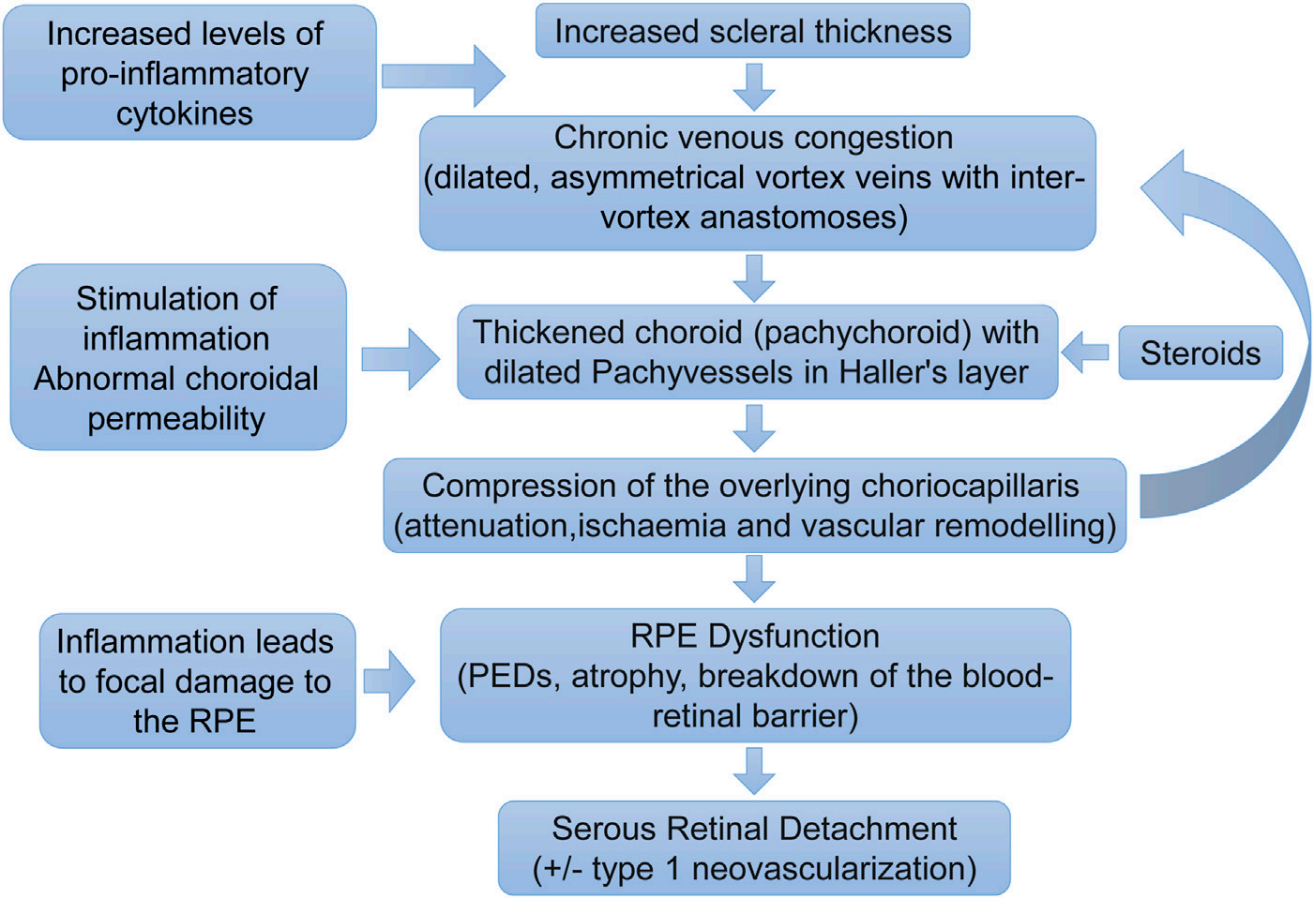
Pathogenesis of central serous chorioretinopathy. A proposed pathogenic pathway for development of central serous chorioretinopathy. Pathology does not necessarily occur in a step-by-step linear fashion. The pathogenesis is likely to be multifactorial with feedback processes (Fung et al., 2023).

In 1967, Gass proposed that CSC was secondary to choroidal hyperpermeability (Gass, 1967). This was subsequently confirmed in intermediate hyperfluorescence in CSC patients undergoing indocyanine green angiography (ICGA) (Hayashi et al., 1986). Theories for this hyperpermeability include RPE degeneration, infection, vascular abnormalities, ischemia, and congestion. Recent studies have shown that choroid vein dilatation, delayed choroid vein filling, eddy vein asymmetry, anastomosis between vortexes, thicker choroid, extrachoroid vessel dilatation (thick blood vessel) in Haller layer, choroid and internal choroid attenuation in Sattler layer, longer choroid vessels with larger diameter and fewer branches (Figure 3).

**FIGURE 4:**
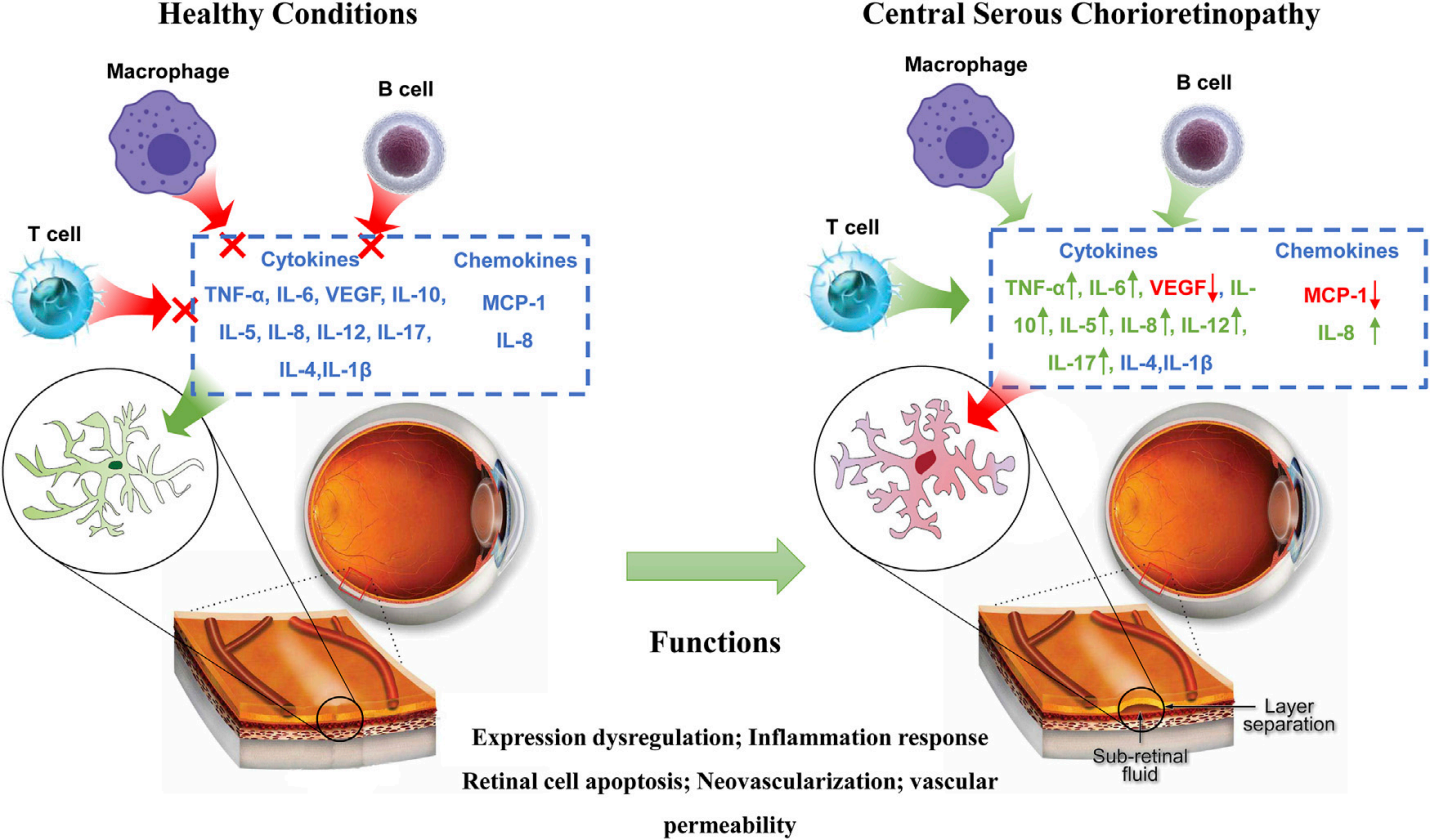
Immune regulation in CSC. Arrows indicate elevated levels or increased activity.

Inflammation serves as a critical element in the pathogenesis of CSC, as exemplified in Figure 4. Although cytokines are commonly implicated in ocular diseases that elicit choroidal abnormalities, their specific role in CSC and underlying mechanisms of action remain uncertain. (Schellevis et al., 2018; Weinstein and Pepple, 2018). In particular, the relationship between endogenous or exogenous steroid exposure and the development of CSC needs further investigation. Studies have shown that steroids can aggravate the progression of CSC. Therefore, steroid-insensitive inflammation is currently considered to be the main mechanism causing CSC inflammation. While some studies have observed heightened levels of pro-inflammatory cytokines, such as IL-6, in CSC patients, the connection between CSC and systemic inflammation remains inconclusive. Nevertheless, the presence of choroidal hyperpermeability and leakage in CSC has spurred additional research into inflammatory markers involved in this pathway (Tittl et al., 1999). In recent years, investigations have delved into serum-based inflammation markers as potential predictors of CSC progression or severity. Notably, alterations in plasma cytokine levels have been observed in CSC patients, featuring a decrease in VEGF expression and an increase in IL-6, IL-10, and IL-12 expression. These changes may reflect the indirect contribution of these factors to the vascular transformations that occur during CSC (Karska-Basta et al., 2021b). Sirakaya et al. explored the correlation between disease states such as diabetic retinopathy and the ratio of monocytes to HDL (Wang et al., 2015; Sirakaya et al., 2020). Additionally, Limon et al. conducted a study on serum fibrinogen/albumin ratios among patients with acute and chronic CSC. Their findings indicated significantly higher fibrinogen/albumin ratios in patients with acute CSC, relative to those with chronic CSC or healthy control subjects (Limon et al., 2022). According to research conducted by Matet et al., patients diagnosed with acute and CSC displayed a decrease in Lipocalin two levels, which is an acute phase reactant known for its properties of both anti-inflammatory and pro-inflammatory effects when compared to individuals who were deemed healthy controls (Matet et al., 2020).

The development of choroidal hyperosmosis in CSC may be attributed to various potential upstream, molecular, and pathological mechanisms, as suggested in a recent study (Yamada-Okahara et al., 2023). These mechanisms may act synergistically to determine the severity of the condition in affected individuals. For instance, compression or congestion of the vortex vein could potentially disrupt choroid function, leading to the breakdown of the retinal pigment epithelium (RPE) barrier, formation of choroidal neovascularization (CNV), and accumulation of subretinal fluid (SRF) (Kiraly et al., 2022). The presence of pro-inflammatory cytokines has been linked to the development of CSC, while activation of mineralocorticoid receptors has been found to cause choroidal vasodilation, resulting in the thickening of the choroid and eventual progression of CSC. Given that CSC can ultimately lead to permanent vision loss in affected individuals, further research must be conducted to better understand the potential underlying causes of this debilitating condition.

## 4 Inflammatory cytokines in CSC

Cytokines are a diverse group of molecules that play crucial roles within the immune system, with each protein possessing a unique structure and specific functions (Baltmr et al., 2014). Among these cytokines, certain molecules such as IL-6 activate downstream responses via the JAK/STAT signaling pathway, while others like IL-1 and IL-17 trigger activation of the NF-κB signaling pathway (Deepak et al., 2022). Certain cytokines play a crucial role in promoting inflammation, including but not limited to IL-1 and TNF-α, commonly referred to as pro-inflammatory cytokines. In contrast, other cytokines like IL-4 and IL-10 function to suppress pro-inflammatory activity, earning them the label of anti-inflammatory cytokines. Proinflammatory cytokines can increase the expression of proinflammatory genes that code for enzymes responsible for producing various molecules such as leukotrienes, platelet activators, NO, and prostaglandins. Additionally, these cytokines are involved in triggering endothelial adhesion molecules, which are pivotal in facilitating the attachment of white blood cells to the surfaces of endothelial cells. As a result, pro-inflammatory cytokines are known to trigger inflammation, tissue damage, and dysfunction within the body. Conversely, anti-inflammatory cytokines work to counteract this process, either by impeding it altogether or by reducing the intensity of the body’s inflammatory response (Dinarello, 2000).

### 4.1 Role of pro-inflammatory cytokines in CSC

TNF-α, a pro-inflammatory cytokine, is synthesized by macrophages, natural killer cells, or T cells, and is closely linked to CSC (Schellevis et al., 2018), which is characterized by tissue inflammation. The cytokine can lead to tissue damage associated with CSC. In a study conducted by Izabella et al., plasma cytokine levels were examined in patients with acute and chronic CSC. The researchers discovered a positive correlation between TNF-α levels and hypertension in chronic CSC (Karska-Basta et al., 2021b). TNF-α stands out as a critical pro-inflammatory cytokine that plays a pivotal role in the development of vasodilation and edema. Through its expression of adhesion molecules, it contributes to the adhesion of leukocytes to epithelial cells, which further exacerbates the inflammatory response. Not only does it regulate blood clotting, but it also adds to oxidative stress at the inflammation site. Additionally, when cytokines become activated, they release intracellular adhesion molecules like ICAM-1 and VCAM-1, which attract monocytes and white blood cells, thereby perpetuating the inflammatory response over a more extended period (Chen et al., 2005). In the context of chronic inflammation, inflammatory cells progressively infiltrate and cause tissue destruction, which only serves to worsen the severity of retinal vascular permeability, vasodilation, and serous retinal detachment in patients suffering from CSC.

IL-6, functioning as a multifaceted inflammatory factor, is closely linked to immune regulation, the augmentation of vascular permeability, and the stimulation of angiogenesis (Semeraro et al., 2015). The crucial involvement of IL-6 in the pathophysiology of both acute and chronic CSC cannot be overstated. In patients with chronic CSC, the upregulation of proinflammatory cytokines, IL-6 and IL-8, was significantly higher than that in patients with acute CSC or PNV (Terao et al., 2018). In cases of chronic CSC, the concentrations of IL-6 and VEGF-A demonstrated a strong correlation with the choroidal hypertonic area, while IL-6 and VEGF concentrations were also found to be correlated with choroidal thickness, along with bFGF and GM-CSF concentrations. Although the correlation between cytokines and the mean thickness of the large choroidal vascular layer was slightly weaker, the trend remained similar (Terao et al., 2018).

There are situations where CSC failed to exhibit any alterations in VEGF levels (Shin and Lim, 2011), however, in certain scenarios, the levels of VEGF experienced a noteworthy downregulation (Jung et al., 2014; Chrząszcz et al., 2021). On the flip side, some researchers have observed a positive correlation between disease progression and the escalation of both VEGF and inflammatory cytokines levels (Jung et al., 2014; Terao et al., 2018). Numerous investigations have indicated that VEGF levels are typically elevated in chronic CSC compared to acute CSC, albeit the disparity between the two is not statistically significant (Lim et al., 2010). It is worth noting that multiple studies have revealed a noteworthy association between the duration of CSC symptoms and VEGF levels. Additionally, VEGF levels are substantially heightened in patients who positively respond to intravitreal injections of bevacizumab (Lim et al., 2010). The expression of VEGF may be markedly elevated in certain instances of CSC, resulting in considerable variability in VEGF levels among various types of CSC (Hara et al., 2023).

IL-5 stands out as a potent inflammatory mediator, renowned for its pivotal role in the immune response. It has a fascinating origin story, tracing back to the quest for a T-cell replacement factor (TRF) responsible for priming B-cells towards antibody production in response to antigens. Researchers eventually unraveled that IL-5 was the elusive TRF, which drove the differentiation of B-cells into antibody-forming cells upon encountering antigens (Takatsu, 1993). The IL-12 family of cytokines takes center stage in modulating T-cell responses, acting as crucial regulators in immune function. The orchestration of these responses is masterfully executed by a triad of immune cells, namely, monocytes, macrophages, and dendritic cells. In response to infections, these immune cells produce a diverse array of IL-12 cytokine family members to fine-tune T-cell responses and maintain immune homeostasis (Gee et al., 2009). The IL-12 cytokine family is a multifaceted entity that plays a critical role in a myriad of biological processes, including cytokine-mediated signaling pathways, and their dysregulation can contribute to the onset of inflammatory diseases, infections, and autoimmune disorders. In patients with acute CSC, there was a significant correlation observed between elevated levels of IL-5, IL-6, and IL-12 and increased mean CT values, implicating the involvement of these cytokines in the pathogenesis of this ocular disease. Meanwhile, in patients with chronic CSC, elevated plasma levels of IL-8, IL-6, and TNF-α were found to be associated with hypertension, shedding light on the potential role of these cytokines in the pathophysiology of chronic CSC and its associated comorbidities. (Karska-Basta et al., 2021b). Moreover, the expression of IL-12, p70, and IL-10 was upregulated (Karska-Basta et al., 2021b). Table 1 displays the plasma cytokine levels of individuals afflicted with both acute and chronic CSC, alongside the control group. Yu et al. constructed a rat model to simulate CSC properties through the injection of aldosterone into the intravitreal area. Their findings revealed a significant upregulation in COX-2, CXCL-1, MMP-2, MMP-9, IL-6, CCL-3, and IL-1β levels, in contrast to the normal control group. Notably, the mRNA levels of TNF-α, IL-10, and CCL-5 remained unaltered (Yu et al., 2022). However, another study that analyzed cytokine expression in the plasma of patients with CSC showed no significant change in IL-1β levels (Karska-Basta et al., 2021b). IL-1β plays a critical role in the development of numerous inflammation-related ailments and is considered one of the primary cytokines involved. While its production and exportation from the cell are essential for the host response and protection against pathogens, it can also worsen damage in cases of chronic illness and acute tissue damage. It is no surprise that there is considerable interest among individuals regarding the mechanisms behind the synthesis and secretion of this protein (Lopez-Castejon and Brough, 2011).

**TABLE 1:** Plasma cytokine levels in patients with acute and chronic central serous chorioretinopathy and controls (Karska-Basta et al., 2021b).

Cytokine (pg/mL)	Control	Acute CSC	Chronic CSC	*p*-value
**TNF-α**	3.26 (2.65–3.79)	3.11 (2.57–3.79)	3.48 (3.03–3.94)	0.429
**VEGF**	**17.26 (8.59–27.10)**	**7.74 (4.23–17.87)**	**7.70 (3.58–12.46)**	**0.029**
**IL-1β**	0.23 (0.19–0.35)	0.27 (0.20–0.36)	0.28 (0.18–0.41)	0.955
**IL-2**	0.70 (0.55–0.93)	0.93 (0.55–1.33)	0.85 (0.70–1.50)	0.150
**IL-4**	2.06 (1.01–5.31)	3.69 (0.51–5.31)	4.23 (2.06–7.43)	0.319
**IL-5**	0.19 (0.15–0.27)	0.25 (0.20–0.30)	0.25 (0.20–0.35)	0.064
**IL-6**	**1.52 (1.10–1.95)**	**2.18 (1.73–2.86)**	**2.29 (1.52–3.56)**	**0.005**
**IL-8**	2.49 (1.68–3.28)	1.68 (1.29–2.12)	1.67 (1.36–2.68)	0.103
**IL-10**	**0.30 (0.23–0.39)**	**0.37 (0.30–0.43)**	**0.37 (0.30–0.51)**	**0.030**
**IL-12 p70**	**2.23 (1.91–2.73)**	**2.73 (2.06–3.46)**	**2.73 (2.39–3.46)**	**0.028**
**GM-CSF**	0.32 (0.25–0.49)	0.25 (0.19–0.32)	0.30 (0.23–0.36)	0.114
**IFN-γ**	1.87 (1.52–2.24)	2.62 (1.52–3.84)	2.06 (1.52–3.43)	0.238

Data are shown as median (interquartile range). Significant correlations are presented in bold.

Comprising of six ligands (IL-17A-F) and five receptors (IL-17RA-RE) (Ruiz de Morales et al., 2020). The IL-17 family holds immense significance in the realm of immunity and inflammation. Amongst these, IL-17A is primarily generated by T-assisted 17 (Th17) cells and functions as a critical mediator of immune and inflammatory responses (Bunte and Beikler, 2019). When IL-17A and IL-17F interact with IL-17RA and IL-17RC, they trigger the activation of both the nuclear factor-κB (NF-κB) and mitogen-activated protein kinase signaling pathways. The result of this activation is the generation and release of inflammatory factors, chemokines, and matrix metalloproteinases (MMPs) - all of which play an instrumental role in the inflammation process. (Taams et al., 2018). Numerous investigations have showcased that exposure to aldosterone can bring about the triggering of signaling pathways such as IL-17 and NF-κB (Sanz-Rosa et al., 2005; Herrada et al., 2010). Nevertheless, the linkage between the IL-17/NF-κB signaling pathway and the thickening of the choroidal layer stimulated by aldosterone is yet to be explored. In rat models of CSC pre-treated with aldosterone, the expression of IL-17A escalated in the afflicted CSC rats in comparison to their healthy counterparts (Yu et al., 2022). The IL-17A/NF-κB pathway is activated and the expression of the IL-17A/NF-κB pathway can be inhibited by prophylactic treatment with melatonin. The outcomes imply that the signaling pathway of IL-17A/NF-κB may play a role in the modulation of melatonin over the choroidal vascular transformations incited by aldosterone.

### 4.2 Role of anti-inflammatory cytokines in CSC

IL-10 plays a crucial role in suppressing inflammatory responses to microbial antigens by acting as an anti-inflammatory cytokine. Its significance lies in its ability to negatively regulate immune responses, thus preventing excessive inflammation that can lead to tissue damage and various pathological conditions. (Rutz and Ouyang, 2016). The behavior of choroidal neovascularization is complex and multidirectional, with immune regulation being just one of its many facets. Recent research conducted by Liu and colleagues explored the levels of various inflammatory factors, including vascular endothelial growth factor (VEGF) and several cytokines (IL-8, IL-10, IP-10, and MCP-1), in patients with different forms of choroidal vascular disease. The study revealed that the levels of angiogenesis index and inflammatory cytokines were significantly elevated in the group with choroidal vascular disease, indicating that inflammation likely contributes to the development of this condition (Liu et al., 2021). The roles of cytokines in CSC as show in Table 2.

**TABLE 2:** The roles of inflammatory cytokines and chemokines in CSC.

Cytokines and chemokines	Mechanism	Function	Article
**TNF-α**	1. Regulates vasodilation and edema 2. Release adhesion molecules 3. Enhanced oxidative stress	Pro-inflammatory	Karska-Basta et al. (2021b)
**IL-6**	1. Choroidal hyperosmosis 2. Regulate VEGF-α expression 3. Regulate bFG and GM-CSF	Pro-inflammatory	Terao et al. (2018)
**IL-5**	1. Upregulated in CSC	Pro-inflammatory	Karska-Basta et al. (2021b)
**IL-12**	1. Upregulated in CSC 2. Regulates T-cell responses	Pro-inflammatory	Karska-Basta et al. (2021b)
**IL-17A**	1. Upregulated in CSC 2.Active the NF-κB pathway	Pro-inflammatory	Yu et al. (2022)
**IL-10**	Negatively modulates the immune response	Anti-inflammatory	Liu et al. (2021)
**IL-8**	1.Upregulated in CSC 2. Active VEGF receptors 3. Increased endothelial cell permeability	Pro-inflammatory	Terao et al. (2018)
**MCP-1**	1. Recruit Monocytes and macrophages 2. Active VEGF and RhoA 3. Increased release of proinflammatory cytokines	Pro-inflammatory	Liu et al. (2021)

## 5 Chemokines

Chemokines are a group of heparin-binding proteins that play a crucial role in guiding white blood cells to sites of inflammation or injury. These small molecules can be categorized into four families based on their distinct structures and functions: CC chemokines, CXC chemokines, CX3C chemokines, and XC chemokines. By binding to specific receptors, chemokines initiate signaling pathways that ultimately result in actin rearrangement, shape modification, and cell locomotion (Charo and Ransohoff, 2006; Hashida et al., 2022).

As the most extensively studied CXC chemokine, IL-8 is recognized for its potent pro-inflammatory properties and thus is tightly regulated and minimally expressed in normal tissues. Macrophages and monocytes release IL-8 when activated, and it plays a crucial role in guiding the targeted migration of basophils, neutrophils, and T cells. The impact of IL-8 on the eye can vary significantly based on its site of action and production. Despite this, one of the most surprising findings about IL-8 is its ability to exhibit angiogenic activity throughout any region of the eye (Ghasemi et al., 2011). Endothelial and glial cells involved in retinal ischemic vasculogenesis are responsible for producing IL-8. Consequently, heightened levels of IL-8 have been observed in the aqueous fluid of individuals with retinal artery occlusion, uveitis, and proliferative diabetic retinopathy (Yuuki et al., 2001; Kramer et al., 2005; Paroli et al., 2007). Patients diagnosed with CSC exhibit choroidal lobular ischemia, choroidal venous congestion, and multizone choroidal vascular permeability during indocyanine angiography. These observations imply the likelihood of a shared RPE or choroidal vascular disorder among individuals with CSC (Petreaca et al., 2007). Furthermore, there is evidence to suggest that VEGF and IL-8 can prompt endothelial cell permeability via transactivation of the VEGF receptor. As a result, it is plausible that individuals with central serous chorioretinopathy may experience an overexpression of both VEGF and IL-8 (Kramer et al., 2005). According to recent studies, patients with chronic central serous chorioretinopathy who also suffer from hypertension tend to have elevated levels of IL-8 and IL-6 in their plasma (Karska-Basta et al., 2021b). In a separate study, Terao et al. discovered upregulation of IL-6, IL-8, and MCP-1 in individuals with acute to chronic CSC. They hypothesize that prolonged subretinal fluid retention may impair RPE function and cause an increase in proinflammatory cytokine expression (Terao et al., 2018).

MCP-1 is a chemokine that falls under the CC family and plays a critical role in the advancement of vascular inflammation associated with diabetic retinopathy. It serves as a potent chemotactic factor, drawing in monocytes and macrophages to the affected site (Taghavi et al., 2019). MCP-1 has been identified as a contributor to vision loss in patients with central serous chorioretinopathy, triggering angiogenesis by stimulating VEGF and activating RhoA (Hong et al., 2005). Furthermore, MCP-1 triggers microglia cell activation, resulting in the release of pro-inflammatory cytokines that can damage retinal neurons and cause optic blood vessel rupture (Dong et al., 2012). In a study conducted by Liu et al., various choroidal neovascularization (CNV) diseases and types were compared to determine the water concentrations of VEGF and other inflammatory factors. Interestingly, their findings showed that MCP-1 expression was downregulated in CSC compared to the control group (Liu et al., 2021). In contrast, Mao et al. did not observe significant differences in the levels of IL-6, IL-8, IL-10, IP-10, and MCP-1 when they compared intraocular cytokines in patients with chronic CSC who had different types of pigment epithelial shedding (PED). They also investigated the relationship between cytokine levels and response to anti-VEGF treatment (Mao et al., 2022). Therefore, the expression of cytokines and chemokines is still different between different subtypes. The roles of chemokines in CSC as show in Table 2.

## 6 Therapies targeting inflammation in CSC

**FIGURE 5:**
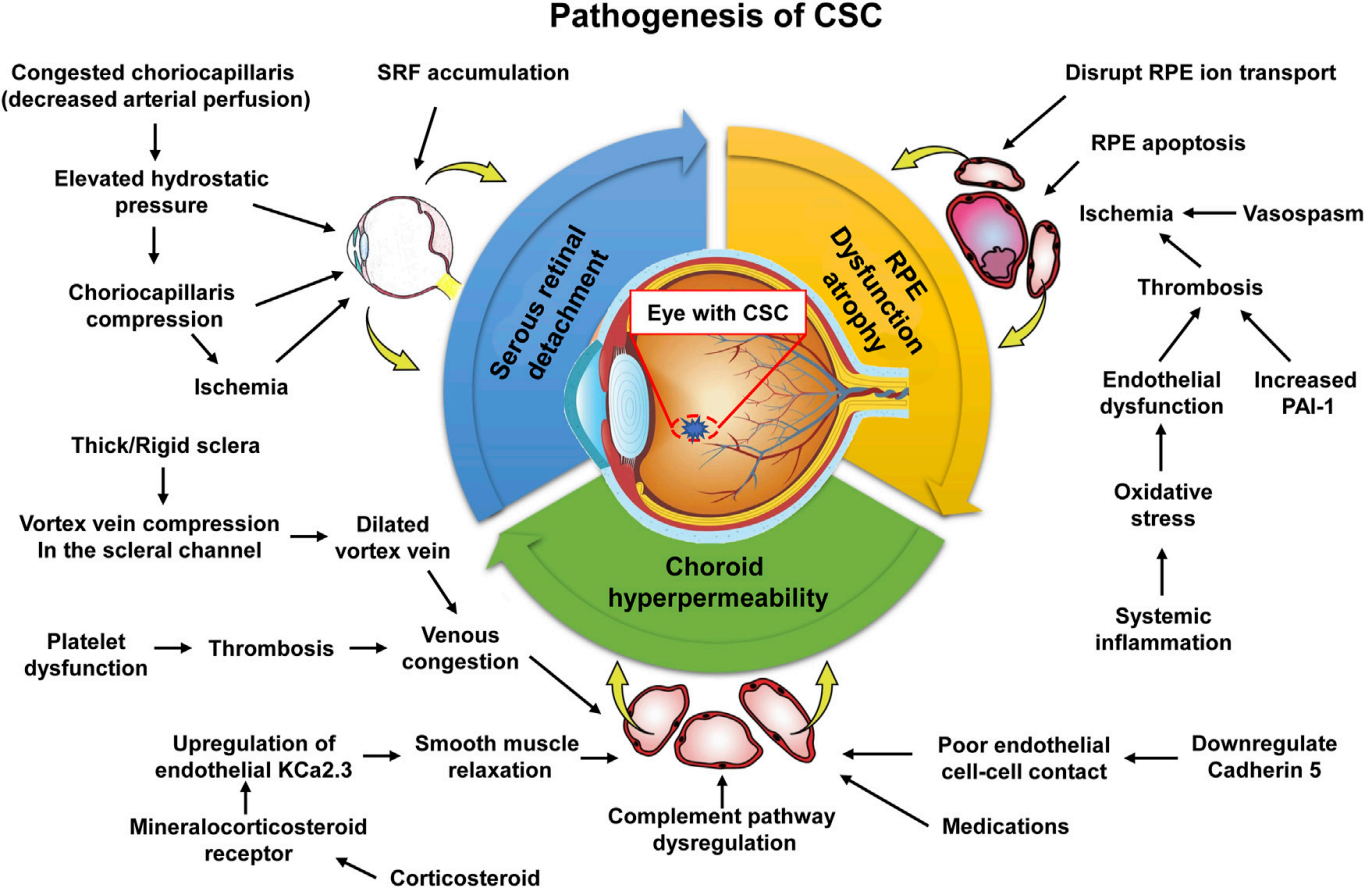
Molecular pathophysiological mechanisms causing serous retinal detachment, pigment epithelial detachment, and choroidal neovascularization in CSC. The upstream mechanisms leading to choroid hyperpermeability include vortex vein compression, corticosteroid receptor activation, and oxidative stress/inflammation.

With the progress in the study of the molecular pathophysiological mechanisms that cause CSC serous retinal detachment, pigment epithelial detachment and choroidal neovascularization, the treatment of CSC has also made encouraging progress. Figure 5 shows the pathophysiological mechanism of CSC. CSC has a good natural course, with spontaneous regression and improved visual function. A high spontaneous response rate is favorable for conservative therapy as a first-line treatment option (Lim et al., 2010). However, in some cases of CSC, due to the continuous serous retinal detachment and macular degeneration sac sample or RPE decompensated, patients may appear progressive loss of vision. An early resolution may offer a potential advantage of reducing the incidence of RPE degeneration in the treated eye. However, since separation occurs and the relationship between symptoms and the specific demands of binocular vision function is unclear, it is difficult to determine the exact benefits (Levine et al., 1989; Loo et al., 2002). Existing interventions include laser photocoagulation, axillary thermal therapy, subliminal micropulse laser, and PDT (van Rijssen et al., 2019). Focus laser and PDT be effective in treating CSC. Focused lasers have been known to cause permanent blind spots that may increase in size as RPE scarring expands, and there is also a risk of choroidal neovascularization (Chan et al., 2003; Colucciello, 2006; Erikitola et al., 2014). In addition, PDT may also have some potential adverse effects such as choroidal hypoperfusion and the development of secondary CNV. However, the efficacy of other treatment options remains largely unclear. A 45-year-old Caucasian female with diagnosis of CSC did not improve on conventional observational approach. She was not willing to proceed with photocoagulation or PDT. An unconventional approach of topical anti-inflammatory (ketorolac, dexamethasone and hydrocortisone) preparation was prescribed. The course of her CSC responded well on this unconventional treatment, but relapsed on cessation or tapering of treatment. After 18 weeks of treatment with a gradual taper, her condition resolved. The present case highlights an alternative but unconventional treatment of CSC with prolonged use of anti-inflammatories (Chong et al., 2012).

In recent years, the treatment of CSC with intravitreal injections of anti-vascular endothelial growth factor (anti-VEGF) has gained popularity (Daruich et al., 2015). Despite this, there is no agreement on the intraocular VEGF levels associated with CSC, nor is there consensus regarding the effectiveness of anti-VEGF therapy for treating CSC. Numerous studies have investigated the involvement of various cytokines, including VEGF, IL-6, IL-8, interferon-induced protein-10 (IP-10), and monocyte chemotactic protein-1 (MCP-1), in the pathogenesis of CSC (Karska-Basta et al., 2021a; Karska-Basta et al., 2021b; Chrząszcz et al., 2021). Lim et al. conducted a study that included 12 patients with CSC and a control group of cataract patients. They measured the levels of vascular endothelial growth factor (VEGF) and IL-8 in CSC patients before a single intravitreal injection of bevacizumab. A study conducted by Lim et al. involving 12 CSC patients and cataract patients as control subjects revealed that there were no significant elevations in VEGF and IL-8 levels in the aqueous humor and plasma of CSC patients. While the use of intravitreal bevacizumab injection in the treatment of CSC is promising, its full efficacy is yet to be established. The effectiveness of anti-VEGF therapy in treating CSC remains to be fully understood (Zhao and Zhang, 2022). Jung et al. reported that patients with a higher sensitivity to intravitreal injections of bevacizumab had significantly elevated levels of VEGF (Jung et al., 2014). These findings imply that elevated levels of VEGF in the eye could potentially respond positively to targeted therapy, indicating that anti-VEGF therapy may be a viable treatment option for CSC or exert its effects through an alternative pathway (Lim et al., 2010). In another research, 88 patients with chronic CSC and 30 controls were compared, and the study showed increased levels of intraocular VEGF in chronic CSC patients. Administration of concept, an anti-VEGF drug, through intravenous injection, improved symptoms and lower levels of inflammatory cytokines in patients with chronic CSC (Mao et al., 2022).

There has been a multitude of studies that have demonstrated the numerous ways in which melatonin can offer protection against endothelial dysfunction, vascular inflammation, and disruption of the blood-retinal barrier (BRB) (Clemson et al., 2017; Shafabakhsh et al., 2019; Gurunathan et al., 2020). Despite the extensive research conducted on melatonin, its impact on CSC (Central Serous Chorioretinopathy), and the exact mechanisms behind its pathogenesis still lack clarity. In an attempt to simulate the characteristics of CSC, Yu et al. (2022) administered intravitreal injection of aldosterone in rats, establishing an experimental model. According to the findings, the timely use of melatonin had a notable impact on suppressing choroidal thickening and vasodilation caused by aldosterone. Additionally, it was observed that the curvature of the choroidal vascular structure was weakened due to a reduction in the expression of KCa2.3, a type of calcium-activated potassium channel. Furthermore, it has been observed that melatonin can safeguard the integrity of the blood-retinal barrier (BRB) and prevent the reduction of tight junction proteins (ZO-1, occludin, and claudin-1) induced by aldosterone in rat models. Moreover, the results demonstrated that the prompt administration of melatonin via intraperitoneal injection effectively suppressed macrophage/microglia infiltration triggered by aldosterone. The levels of several inflammation-related factors, including IL-6, IL-1β, cyclooxygenase-2, chemokine C-C motif ligand 3, C-X-C motif ligand 1, as well as matrix metalloproteinases MMP-2 and MMP-9, were significantly reduced as well. Furthermore, it was found that the administration of aldosterone via intravitreal injection triggered the activation of the IL-17A/nuclear factor-κB signaling pathway, which was effectively inhibited in the eyes that were pre-treated with melatonin. These results strongly indicate that melatonin has great potential as a safe treatment strategy for individuals suffering from CSC.

Eplerenone, another aldosterone antagonist, has also shown potential to treat CSC, and Cioboata et al. reported a 46-year-old patient with unilateral recurrent CSC who, after initial medical therapy (oral carbonic anhydrase inhibitor, oral antihistamines, and nonsteroidal anti-inflammatory drugs-systemic and topical), with an oral aldosterone antagonist-Eplerenone (Inspra), resulted in significant anatomic and visual improvements (Cioboata et al., 2016).

Takayama et al. (2020) report the effectiveness of adalimumab was investigated in a patient with chronic Vogt-Koyanagi-Harada (VKH) disease, which had proven resistant to standard treatment with corticosteroids and immunosuppressive therapy. Additionally, the patient had developed complications due to CSC. The individual in question exhibited CSC in both eyes as a result of recurring ocular inflammation, as well as systemic complications such as diabetes, bone weakness, and moon-face. It was not possible to gradually reduce the usage of systemic corticosteroids in this case. The diagnosis was confirmed as chronic VKH resistance complicated by glucocorticoid-induced CSC. Since bilateral CSC and adverse effects related to corticosteroids could be aggravated by continuing corticosteroid therapy, systemic oral corticosteroid (0.2 mg/kg/day) was immediately tapered and adalimumab (40 mg every 2 weeks) was initiated. After initiation of adalimumab therapy, bilateral ocular inflammation, PED in the right eye, and bullous RD in the left eye were reduced gradually without any adverse effect. One month from starting adalimumab BCVA was 20/25 in the right eye and 20/50 in the left eye, no inflammation was present in the anterior chamber and vitreous, and PED in the right eye as well as bullous RD in left one were reduced. Therefore, adalimumab shows great promise as a safe treatment option for individuals with CSC.

## 7 Concluding remarks and future perspectives

At present, the pathogenesis of CSC is not clear. However, choroidal hyperosmosis may be related to the abnormal choroidal circulation, especially the attenuation of the chorionic membrane, and the destruction of the RPE monomolecular layer is also thought to lead to the accumulation of SRF (Jeon et al., 2022). CSC is currently considered to be one of the choroidal-thickened diseases, characterized by a thickened choroidal blood vessel (Chen and Chen, 2020). Clinicopathological studies with histology of RPE, choroid, and sclera can improve our understanding of this disease (Fusi-Rubiano et al., 2020; Fung et al., 2023). There are various plausible mechanisms at play, which could potentially contribute to the hyperpermeability of choroidal vasculature in CSC. These mechanisms may operate at different levels, ranging from upstream signaling pathways and molecular events to more complex pathological processes (Kanda et al., 2022; Zarnegar et al., 2023). The progression of CSC is often linked with the presence of pro-inflammatory cytokines. Additionally, the activation of mineral corticosteroid receptors may induce the expansion of choroidal blood vessels, ultimately leading to the thickening of the choroid and exacerbating CSC (Jung et al., 2014; Hua et al., 2021). One potential avenue for treating CSC is to utilize anti-inflammatory methods that focus on specific molecular markers. In recent times, it has been acknowledged that inflammation plays a crucial role in the advancement and onset of CSC. Hence, targeting inflammation through molecular markers could be a promising treatment strategy for CSC(Jain et al., 2022). The inflammatory process of CSC involves a dynamic interplay between cytokines, chemokines, and adhesion molecules. These three molecular players work in tandem and cannot be considered in isolation, as they form a complex network that drives the progression of CSC lesions. Their interactions are crucial to the pathological process and highlight the intricate nature of inflammation in CSC (Daruich et al., 2018). Cytokines are divided into proinflammatory cytokines and anti-inflammatory cytokines according to their functions (Opal and DePalo, 2000). Upon the onset of inflammation, there is an increase in the production of pro-inflammatory cytokines, which play a pivotal role in amplifying the inflammatory response via several pathways. These pathways include the triggering of retinal cell apoptosis and the induction of angiogenesis, which further propagate the inflammatory cascade (Watson et al., 2016). N addition to pro-inflammatory cytokines, anti-inflammatory cytokines may also be present in increased levels to counterbalance the protective effect of the inflammatory response. Chemokines play a crucial role as cellular transporters, facilitating the recruitment of circulating white blood cells to the site of inflammation. Adhesion molecules also come into play, aiding in the attachment of white blood cells to the site of inflammation. Despite these molecular intricacies, the complex pathological mechanism underlying the involvement of inflammatory factors in CSC has yet to be fully understood and requires further in-depth investigation. In the coming years, there is great potential for continued exploration of the immunoinflammatory mechanism underlying CSC. Such efforts promise to advance our understanding of the biological function of CSC, paving the way for the development of targeted therapies. Indeed, these findings underscore the clinical significance of novel treatments that focus on addressing inflammatory responses in the management of CSC. Ultimately, this research will facilitate the translation of recent experimental discoveries into clinical practice, benefiting patients in need.

Despite the significant strides we’ve made in comprehending CSC, there’s still a wealth of knowledge yet to be uncovered regarding this affliction of the choroid. Given the debilitating nature of CSC and its propensity to cause permanent blindness, we must persist in our exploration of the diverse potential root causes of the disease. As our comprehension of the molecular underpinnings of CSC expands, we may be more proficient in categorizing individuals who are more vulnerable to the disease and assessing tailored therapeutic approaches for their medical management.

## References

[B1] Baltmr A. Lightman S. Tomkins-Netzer O. (2014). Examining the choroid in ocular inflammation: a focus on enhanced depth imaging. J. Ophthalmol. 2014, 459136. 10.1155/2014/459136 25024846 PMC4082870

[B2] Baran N. V. Gürlü V. P. Esgin H. (2005). Long-term macular function in eyes with central serous chorioretinopathy. Clin. Exp. Ophthalmol. 33 (4), 369–372. 10.1111/j.1442-9071.2005.01027.x 16033348

[B3] Berger L. Bühler V. Yzer S. (2021). Central serous chorioretinopathy - an overview. Klin. Monbl Augenheilkd 238 (9), 971–979. 10.1055/a-1531-5605 34416788

[B4] Breukink M. B. Dingemans A. J. den Hollander A. I. Keunen J. E. MacLaren R. E. Fauser S. (2017). Chronic central serous chorioretinopathy: long-term follow-up and vision-related quality of life. Clin. Ophthalmol. 11, 39–46. 10.2147/opth.s115685 28053499 PMC5189979

[B5] Bunte K. Beikler T. (2019). Th17 cells and the IL-23/IL-17 Axis in the pathogenesis of periodontitis and immune-mediated inflammatory diseases. Int. J. Mol. Sci. 20 (14), 3394. 10.3390/ijms20143394 31295952 PMC6679067

[B6] Chan W. M. Lam D. S. Lai T. Y. Tam B. S. Liu D. T. Chan C. K. (2003). Choroidal vascular remodelling in central serous chorioretinopathy after indocyanine green guided photodynamic therapy with verteporfin: a novel treatment at the primary disease level. Br. J. Ophthalmol. 87 (12), 1453–1458. 10.1136/bjo.87.12.1453 14660450 PMC1920573

[B7] Charo I. F. Ransohoff R. M. (2006). The many roles of chemokines and chemokine receptors in inflammation. N. Engl. J. Med. 354 (6), 610–621. 10.1056/NEJMra052723 16467548

[B8] Chen W. Esselman W. J. Jump D. B. Busik J. V. (2005). Anti-inflammatory effect of docosahexaenoic acid on cytokine-induced adhesion molecule expression in human retinal vascular endothelial cells. Invest. Ophthalmol. Vis. Sci. 46 (11), 4342–4347. 10.1167/iovs.05-0601 16249517 PMC1378111

[B9] Chen Y. C. Chen S. N. (2020). Three-year follow-up of choroidal neovascularisation in eyes of chronic central serous chorioretinopathy. Br. J. Ophthalmol. 104 (11), 1561–1566. 10.1136/bjophthalmol-2019-315302 32051140

[B10] Cheung C. M. G. Lee W. K. Koizumi H. Dansingani K. Lai T. Y. Y. Freund K. B. (2019). Pachychoroid disease. Eye (Lond) 33 (1), 14–33. 10.1038/s41433-018-0158-4 29995841 PMC6328576

[B11] Chhablani J. Cohen F. B. (2020). Multimodal imaging-based central serous chorioretinopathy classification. Ophthalmol. Retina 4 (11), 1043–1046. 10.1016/j.oret.2020.07.026 33131671

[B12] Chong C. F. Yang D. Pham T. Q. Liu H. (2012). A novel treatment of central serous chorioretinopathy with topical anti-inflammatory therapy. BMJ Case Rep. 2012, bcr2012006970. 10.1136/bcr-2012-006970 PMC4544367 22949003

[B13] Chrząszcz M. Pociej-Marciak W. Żuber-Łaskawiec K. Romanowska-Dixon B. Sanak M. Michalska-Małecka K. (2021). Changes in plasma VEGF and PEDF levels in patients with central serous chorioretinopathy. Med. Kaunas. 57 (10), 1063. 10.3390/medicina57101063 PMC8540423 34684100

[B14] Cioboata M. Alexandrescu C. Hopinca C. A. Pienaru M. C. Merticariu A. Schmitzer S. (2016). A new treatment approach - eplerenone - in central serous chorioretinopathy - case report. J. Med. Life 9 (1), 92–94. 27713772 PMC5052491

[B15] Clemson C. M. Yost J. Taylor A. W. (2017). The role of alpha-MSH as a modulator of ocular immunobiology exemplifies mechanistic differences between melanocortins and steroids. Ocul. Immunol. Inflamm. 25 (2), 179–189. 10.3109/09273948.2015.1092560 26807874 PMC5769144

[B16] Colucciello M. (2006). Choroidal neovascularization complicating photodynamic therapy for central serous retinopathy. Retina 26 (2), 239–242. 10.1097/00006982-200602000-00027 16467692

[B17] Daruich A. Matet A. Behar-Cohen F. (2017a). Central serous chorioretinopathy. Dev. Ophthalmol. 58, 27–38. 10.1159/000455267 28351043

[B18] Daruich A. Matet A. Dirani A. Bousquet E. Zhao M. Farman N. (2015). Central serous chorioretinopathy: recent findings and new physiopathology hypothesis. Prog. Retin Eye Res. 48, 82–118. 10.1016/j.preteyeres.2015.05.003 26026923

[B19] Daruich A. Matet A. Marchionno L. De Azevedo J. D. Ambresin A. Mantel I. (2017b). Acute central serous chorioretinopathy: factors influencing episode duration. Retina 37 (10), 1905–1915. 10.1097/iae.0000000000001443 28067724 PMC5642321

[B20] Daruich A. Matet A. Moulin A. Kowalczuk L. Nicolas M. Sellam A. (2018). Mechanisms of macular edema: beyond the surface. Prog. Retin Eye Res. 63, 20–68. 10.1016/j.preteyeres.2017.10.006 29126927

[B21] Deepak H. B. Prince S. E. Deshpande P. (2022). Effect of baricitinib in regulating programmed death 1 and ligand programmed cell death ligand 1 through JAK/STAT pathway in psoriasis. Indian J. Pharmacol. 54 (3), 183–193. 10.4103/ijp.ijp_1089_20 35848689 PMC9396682

[B22] Dinarello C. A. (2000). Proinflammatory cytokines. Chest 118 (2), 503–508. 10.1378/chest.118.2.503 10936147

[B23] Dong N. Li X. Xiao L. Yu W. Wang B. Chu L. (2012). Upregulation of retinal neuronal MCP-1 in the rodent model of diabetic retinopathy and its function *in vitro* . Invest. Ophthalmol. Vis. Sci. 53 (12), 7567–7575. 10.1167/iovs.12-9446 23010641

[B24] Erikitola O. C. Crosby-Nwaobi R. Lotery A. J. Sivaprasad S. (2014). Photodynamic therapy for central serous chorioretinopathy. Eye (Lond) 28 (8), 944–957. 10.1038/eye.2014.134 24946843 PMC4135258

[B25] Fung A. T. Yang Y. Kam A. W. (2023). Central serous chorioretinopathy: a review. Clin. Exp. Ophthalmol. 51, 243–270. 10.1111/ceo.14201 36597282

[B26] Fusi-Rubiano W. Saedon H. Patel V. Yang Y. C. (2020). Oral medications for central serous chorioretinopathy: a literature review. Eye (Lond) 34 (5), 809–824. 10.1038/s41433-019-0568-y 31527760 PMC7182569

[B27] Gass J. D. (1967). Pathogenesis of disciform detachment of the neuroepithelium. Am. J. Ophthalmol. 63 (3), 1–139. 6019308

[B28] Gawęcki M. Jaszczuk A. Grzybowski A. (2020). Short term presence of subretinal fluid in central serous chorioretinopathy affects retinal thickness and function. J. Clin. Med. 9 (11), 3429. 10.3390/jcm9113429 33114519 PMC7692782

[B29] Gawęcki M. Jaszczuk-Maciejewska A. Jurska-Jaśko A. Kneba M. Grzybowski A. (2019). Impairment of visual acuity and retinal morphology following resolved chronic central serous chorioretinopathy. BMC Ophthalmol. 19 (1), 160. 10.1186/s12886-019-1171-5 31345183 PMC6659242

[B30] Gee K. Guzzo C. Che Mat N. F. Ma W. Kumar A. (2009). The IL-12 family of cytokines in infection, inflammation and autoimmune disorders. Inflamm. Allergy Drug Targets 8 (1), 40–52. 10.2174/187152809787582507 19275692

[B31] Ghasemi H. Ghazanfari T. Yaraee R. Faghihzadeh S. Hassan Z. M. (2011). Roles of IL-8 in ocular inflammations: a review. Ocul. Immunol. Inflamm. 19 (6), 401–412. 10.3109/09273948.2011.618902 22106907

[B32] Gilbert C. M. Owens S. L. Smith P. D. Fine S. L. (1984). Long-term follow-up of central serous chorioretinopathy. Br. J. Ophthalmol. 68 (11), 815–820. 10.1136/bjo.68.11.815 6541945 PMC1040477

[B33] Gurunathan S. Kang M. H. Kim J. H. (2020). Role and therapeutic potential of melatonin in the central nervous system and cancers. Cancers (Basel) 12 (6), 1567. 10.3390/cancers12061567 32545820 PMC7352348

[B34] Hara C. Wakabayashi T. Sayanagi K. Nishida K. (2023). Refractory age-related macular degeneration due to concurrent central serous chorioretinopathy in previously well-controlled eyes. Pharm. (Basel) 16 (1), 89. 10.3390/ph16010089 PMC9864072 36678586

[B35] Hashida N. Asao K. Hara C. Quantock A. J. Saita R. Kurakami H. (2022). Mitochondrial dna as a biomarker for acute central serous chorioretinopathy: a case-control study. Front. Med. (Lausanne) 9, 938600. 10.3389/fmed.2022.938600 35801206 PMC9253465

[B36] Hayashi K. Hasegawa Y. Tokoro T. (1986). Indocyanine green angiography of central serous chorioretinopathy. Int. Ophthalmol. 9 (1), 37–41. 10.1007/bf00225936 3721709

[B37] Herrada A. A. Contreras F. J. Marini N. P. Amador C. A. González P. A. Cortés C. M. (2010). Aldosterone promotes autoimmune damage by enhancing Th17-mediated immunity. J. Immunol. 184 (1), 191–202. 10.4049/jimmunol.0802886 19949098

[B38] Hong K. H. Ryu J. Han K. H. (2005). Monocyte chemoattractant protein-1-induced angiogenesis is mediated by vascular endothelial growth factor-A. Blood 105 (4), 1405–1407. 10.1182/blood-2004-08-3178 15498848

[B39] Hua R. Duan J. Zhang M. (2021). Pachychoroid spectrum disease: underlying pathology, classification, and phenotypes. Curr. Eye Res. 46 (10), 1437–1448. 10.1080/02713683.2021.1942073 34114902

[B40] Jain M. Mohan S. van Dijk E. H. C. (2022). Central serous chorioretinopathy: pathophysiology, systemic associations, and a novel etiological classification. Taiwan J. Ophthalmol. 12 (4), 381–393. 10.4103/2211-5056.362601 36660127 PMC9843580

[B41] Jeon S. H. Kim M. Lee J. Roh Y. J. (2022). The effect of selective retina therapy for bevacizumab-resistant chronic central serous chorioretinopathy. Ophthalmologica 245 (1), 91–100. 10.1159/000520187 34649253

[B42] Jung S. H. Kim K. A. Sohn S. W. Yang S. J. (2014). Cytokine levels of the aqueous humour in central serous chorioretinopathy. Clin. Exp. Optom. 97 (3), 264–269. 10.1111/cxo.12125 24417755

[B43] Kanda P. Gupta A. Gottlieb C. Karanjia R. Coupland S. G. Bal M. S. (2022). Pathophysiology of central serous chorioretinopathy: a literature review with quality assessment. Eye (Lond) 36 (5), 941–962. 10.1038/s41433-021-01808-3 34654892 PMC9046392

[B44] Karska-Basta I. Pociej-Marciak W. Chrząszcz M. Kubicka-Trząska A. Dębicka-Kumela M. Gawęcki M. (2021a). Imbalance in the levels of angiogenic factors in patients with acute and chronic central serous chorioretinopathy. J. Clin. Med. 10 (5), 1087. 10.3390/jcm10051087 33807809 PMC7961803

[B45] Karska-Basta I. Pociej-Marciak W. Chrząszcz M. Kubicka-Trząska A. Romanowska-Dixon B. Sanak M. (2021b). Altered plasma cytokine levels in acute and chronic central serous chorioretinopathy. Acta Ophthalmol. 99 (2), e222–e231. 10.1111/aos.14547 32701204 PMC7984262

[B46] Kiraly P. Smrekar J. Jaki Mekjavić P. (2022). Morphological parameters predicting subthreshold micropulse laser effectiveness in central serous chorioretinopathy. Lasers Med. Sci. 37 (8), 3129–3136. 10.1007/s10103-022-03574-4 35579726

[B47] Kitzmann A. S. Pulido J. S. Diehl N. N. Hodge D. O. Burke J. P. (2008). The incidence of central serous chorioretinopathy in Olmsted County, Minnesota, 1980-2002. Ophthalmology 115 (1), 169–173. 10.1016/j.ophtha.2007.02.032 18166410

[B48] Klein M. L. Van Buskirk E. M. Friedman E. Gragoudas E. Chandra S. (1974). Experience with nontreatment of central serous choroidopathy. Arch. Ophthalmol. 91 (4), 247–250. 10.1001/archopht.1974.03900060257001 4621147

[B49] Kramer M. Goldenberg-Cohen N. Axer-Siegel R. Weinberger D. Cohen Y. Monselise Y. (2005). Inflammatory reaction in acute retinal artery occlusion: cytokine levels in aqueous humor and serum. Ocul. Immunol. Inflamm. 13 (4), 305–310. 10.1080/09273940590950990 16159722

[B50] Levine R. Brucker A. J. Robinson F. (1989). Long-term follow-up of idiopathic central serous chorioretinopathy by fluorescein angiography. Ophthalmology 96 (6), 854–859. 10.1016/s0161-6420(89)32810-7 2740080

[B51] Lim J. W. Kim M. U. Shin M. C. (2010). Aqueous humor and plasma levels of vascular endothelial growth factor and interleukin-8 in patients with central serous chorioretinopathy. Retina 30 (9), 1465–1471. 10.1097/IAE.0b013e3181d8e7fe 20526231

[B52] Limon U. Bozkurt E. Bulut S. Ilkay B. Akçay S. (2022). Elevated serum fibrinogen/albumin ratios in patients with acute central serous chorioretinopathy. Eur. J. Ophthalmol. 32 (3), 1735–1742. 10.1177/11206721221089773 35306912

[B53] Liu C. Zhang S. Deng X. Chen Y. Shen L. Hu L. (2021). Comparison of intraocular cytokine levels of choroidal neovascularization secondary to different retinopathies. Front. Med. (Lausanne) 8, 783178. 10.3389/fmed.2021.783178 34993212 PMC8725795

[B54] Loo R. H. Scott I. U. Flynn H. W. Jr. Gass J. D. Murray T. G. Lewis M. L. (2002). Factors associated with reduced visual acuity during long-term follow-up of patients with idiopathic central serous chorioretinopathy. Retina 22 (1), 19–24. 10.1097/00006982-200202000-00004 11884873

[B55] Lopez-Castejon G. Brough D. (2011). Understanding the mechanism of IL-1β secretion. Cytokine Growth Factor Rev. 22 (4), 189–195. 10.1016/j.cytogfr.2011.10.001 22019906 PMC3714593

[B56] Mao J. Zhang C. Zhang S. Liu C. Chen N. Tao J. (2022). Predictors of anti-VEGF efficacy in chronic central serous chorioretinopathy based on intraocular cytokine levels and pigment epithelium detachment subtypes. Acta Ophthalmol. 100 (7), e1385–e1394. 10.1111/aos.15109 35122421

[B57] Matet A. Daruich A. Zola M. Behar-Cohen F. (2018). Risk factors for recurrences of central serous chorioretinopathy. Retina 38 (7), 1403–1414. 10.1097/iae.0000000000001729 28570485

[B58] Matet A. Jaworski T. Bousquet E. Canonica J. Gobeaux C. Daruich A. (2020). Lipocalin 2 as a potential systemic biomarker for central serous chorioretinopathy. Sci. Rep. 10 (1), 20175. 10.1038/s41598-020-77202-y 33214636 PMC7677530

[B59] Mrejen S. Balaratnasingam C. Kaden T. R. Bottini A. Dansingani K. Bhavsar K. V. (2019). Long-term visual outcomes and causes of vision loss in chronic central serous chorioretinopathy. Ophthalmology 126 (4), 576–588. 10.1016/j.ophtha.2018.12.048 30659849

[B60] Negi A. Marmor M. F. (1984). Experimental serous retinal detachment and focal pigment epithelial damage. Arch. Ophthalmol. 102 (3), 445–449. 10.1001/archopht.1984.01040030359038 6703994

[B61] Nicholson B. P. Atchison E. Idris A. A. Bakri S. J. (2018). Central serous chorioretinopathy and glucocorticoids: an update on evidence for association. Surv. Ophthalmol. 63 (1), 1–8. 10.1016/j.survophthal.2017.06.008 28673727

[B62] Opal S. M. DePalo V. A. (2000). Anti-inflammatory cytokines. Chest 117 (4), 1162–1172. 10.1378/chest.117.4.1162 10767254

[B63] Paroli M. P. Teodori C. D'Alessandro M. Mariani P. Iannucci G. Paroli M. (2007). Increased vascular endothelial growth factor levels in aqueous humor and serum of patients with quiescent uveitis. Eur. J. Ophthalmol. 17 (6), 938–942. 10.1177/112067210701700611 18050120

[B64] Pauleikhoff L. Agostini H. Lange C. (2021). Central serous chorioretinopathy. Ophthalmologe 118 (9), 967–980. 10.1007/s00347-021-01376-7 33861376

[B65] Petreaca M. L. Yao M. Liu Y. Defea K. Martins-Green M. (2007). Transactivation of vascular endothelial growth factor receptor-2 by interleukin-8 (IL-8/CXCL8) is required for IL-8/CXCL8-induced endothelial permeability. Mol. Biol. Cell 18 (12), 5014–5023. 10.1091/mbc.e07-01-0004 17928406 PMC2096609

[B66] Ruiz de Morales J. M. G. Puig L. Daudén E. Cañete J. D. Pablos J. L. Martín A. O. (2020). Critical role of interleukin (IL)-17 in inflammatory and immune disorders: an updated review of the evidence focusing in controversies. Autoimmun. Rev. 19 (1), 102429. 10.1016/j.autrev.2019.102429 31734402

[B67] Rutz S. Ouyang W. (2016). Regulation of interleukin-10 expression. Adv. Exp. Med. Biol. 941, 89–116. 10.1007/978-94-024-0921-5_5 27734410

[B68] Sanz-Rosa D. Cediel E. de las Heras N. Miana M. Balfagón G. Lahera V. (2005). Participation of aldosterone in the vascular inflammatory response of spontaneously hypertensive rats: role of the NFkappaB/IkappaB system. J. Hypertens. 23 (6), 1167–1172. 10.1097/01.hjh.0000170379.08214.5a 15894892

[B69] Schellevis R. L. van Dijk E. H. C. Breukink M. B. Altay L. Bakker B. Koeleman B. P. C. (2018). Role of the complement system in chronic central serous chorioretinopathy: a genome-wide association study. JAMA Ophthalmol. 136 (10), 1128–1136. 10.1001/jamaophthalmol.2018.3190 30073298 PMC6233836

[B70] Semeraro F. Cancarini A. dell'Omo R. Rezzola S. Romano M. R. Costagliola C. (2015). Diabetic retinopathy: vascular and inflammatory disease. J. Diabetes Res. 2015, 582060. 10.1155/2015/582060 26137497 PMC4475523

[B71] Shafabakhsh R. Reiter R. J. Mirzaei H. Teymoordash S. N. Asemi Z. (2019). Melatonin: a new inhibitor agent for cervical cancer treatment. J. Cell Physiol. 234 (12), 21670–21682. 10.1002/jcp.28865 31131897

[B72] Shin M. C. Lim J. W. (2011). Concentration of cytokines in the aqueous humor of patients with central serous chorioretinopathy. Retina 31 (9), 1937–1943. 10.1097/IAE.0b013e31820a6a17 21478806

[B73] Singh S. R. Matet A. van Dijk E. H. C. Daruich A. Fauser S. Yzer S. (2019). Discrepancy in current central serous chorioretinopathy classification. Br. J. Ophthalmol. 103 (6), 737–742. 10.1136/bjophthalmol-2018-312435 30002069

[B74] Sirakaya E. Duru Z. Kuçuk B. Duru N. (2020). Monocyte to high-density lipoprotein and neutrophil-to-lymphocyte ratios in patients with acute central serous chorioretinopathy. Indian J. Ophthalmol. 68 (5), 854–858. 10.4103/ijo.IJO_1327_19 32317461 PMC7350498

[B75] Spiers J. G. Chen H. J. Sernia C. Lavidis N. A. (2014). Activation of the hypothalamic-pituitary-adrenal stress axis induces cellular oxidative stress. Front. Neurosci. 8, 456. 10.3389/fnins.2014.00456 25646076 PMC4298223

[B76] Taams L. S. Steel K. J. A. Srenathan U. Burns L. A. Kirkham B. W. (2018). IL-17 in the immunopathogenesis of spondyloarthritis. Nat. Rev. Rheumatol. 14 (8), 453–466. 10.1038/s41584-018-0044-2 30006601

[B77] Taban M. Boyer D. S. Thomas E. L. Taban M. (2004). Chronic central serous chorioretinopathy: photodynamic therapy. Am. J. Ophthalmol. 137 (6), 1073–1080. 10.1016/j.ajo.2004.01.043 15183792

[B78] Taghavi Y. Hassanshahi G. Kounis N. G. Koniari I. Khorramdelazad H. (2019). Monocyte chemoattractant protein-1 (MCP-1/CCL2) in diabetic retinopathy: latest evidence and clinical considerations. J. Cell Commun. Signal 13 (4), 451–462. 10.1007/s12079-018-00500-8 30607767 PMC6946768

[B79] Takatsu K. (1993). Cytokine and inflammation: role of IL-5 and its receptor system in inflammation. Nihon Yakurigaku Zasshi 102 (5), 301–312. 10.1254/fpj.102.301 8244211

[B80] Takayama K. Obata H. Takeuchi M. (2020). Efficacy of adalimumab for chronic vogt-koyanagi-harada disease refractory to conventional corticosteroids and immunosuppressive therapy and complicated by central serous chorioretinopathy. Ocul. Immunol. Inflamm. 28 (3), 509–512. 10.1080/09273948.2019.1603312 31268769

[B81] Teke M. Y. Elgin U. Nalcacioglu-Yuksekkaya P. Sen E. Ozdal P. Ozturk F. (2014). Comparison of autofluorescence and optical coherence tomography findings in acute and chronic central serous chorioretinopathy. Int. J. Ophthalmol. 7 (2), 350–354. 10.3980/j.issn.2222-3959.2014.02.29 24790884 PMC4003096

[B82] Terao N. Koizumi H. Kojima K. Yamagishi T. Nagata K. Kitazawa K. (2018). Association of upregulated angiogenic cytokines with choroidal abnormalities in chronic central serous chorioretinopathy. Invest. Ophthalmol. Vis. Sci. 59 (15), 5924–5931. 10.1167/iovs.18-25517 30551200

[B83] Tittl M. K. Spaide R. F. Wong D. Pilotto E. Yannuzzi L. A. Fisher Y. L. (1999). Systemic findings associated with central serous chorioretinopathy. Am. J. Ophthalmol. 128 (1), 63–68. 10.1016/s0002-9394(99)00075-6 10482095

[B84] van Rijssen T. J. van Dijk E. H. C. Yzer S. Ohno-Matsui K. Keunen J. E. E. Schlingemann R. O. (2019). Central serous chorioretinopathy: towards an evidence-based treatment guideline. Prog. Retin Eye Res. 73, 100770. 10.1016/j.preteyeres.2019.07.003 31319157

[B85] Venkatesh R. Pereira A. Jayadev C. Prabhu V. Aseem A. Jain K. (2020). Oral eplerenone versus observation in the management of acute central serous chorioretinopathy: a prospective, randomized comparative study. Pharm. (Basel) 13 (8), 170. 10.3390/ph13080170 PMC7463844 32751370

[B86] Wang M. Munch I. C. Hasler P. W. Prünte C. Larsen M. (2008). Central serous chorioretinopathy. Acta Ophthalmol. 86 (2), 126–145. 10.1111/j.1600-0420.2007.00889.x 17662099

[B87] Wang R. T. Zhang J. R. Li Y. Liu T. Yu K. J. (2015). Neutrophil-Lymphocyte ratio is associated with arterial stiffness in diabetic retinopathy in type 2 diabetes. J. Diabetes Complicat. 29 (2), 245–249. 10.1016/j.jdiacomp.2014.11.006 25483847

[B88] Watson E. C. Koenig M. N. Grant Z. L. Whitehead L. Trounson E. Dewson G. (2016). Apoptosis regulates endothelial cell number and capillary vessel diameter but not vessel regression during retinal angiogenesis. Development 143 (16), 2973–2982. 10.1242/dev.137513 27471260

[B89] Weinstein J. E. Pepple K. L. (2018). Cytokines in uveitis. Curr. Opin. Ophthalmol. 29 (3), 267–274. 10.1097/icu.0000000000000466 29521875 PMC7199509

[B90] Yamada-Okahara N. Kyo A. Hirayama K. Yamamoto M. Kohno T. Honda S. (2023). Practical treatment options for persistent central serous chorioretinopathy and early visual and anatomical outcomes. Jpn. J. Ophthalmol. 67, 295–300. 10.1007/s10384-023-00978-9 36867256

[B91] Yannuzzi L. A. (2010). Central serous chorioretinopathy: a personal perspective. Am. J. Ophthalmol. 149 (3), 361–363. 10.1016/j.ajo.2009.11.017 20172062

[B92] Yavaş G. F. Küsbeci T. Kaşikci M. Günay E. Doğan M. Unlü M. (2014). Obstructive sleep apnea in patients with central serous chorioretinopathy. Curr. Eye Res. 39 (1), 88–92. 10.3109/02713683.2013.824986 24047212

[B93] You E. L. Hébert M. Jin T. S. Bourgault S. Caissie M. Tourville É. (2023). Comparing interventions for chronic central serous chorioretinopathy: a network meta-analysis. Surv. Ophthalmol. 68, 601–614. 10.1016/j.survophthal.2023.03.001 36931437

[B94] Yu S. Cui K. Wu P. Wu B. Lu X. Huang R. (2022). Melatonin prevents experimental central serous chorioretinopathy in rats. J. Pineal Res. 73 (1), e12802. 10.1111/jpi.12802 35436360

[B95] Yuuki T. Kanda T. Kimura Y. Kotajima N. Tamura J. Kobayashi I. (2001). Inflammatory cytokines in vitreous fluid and serum of patients with diabetic vitreoretinopathy. J. Diabetes Complicat. 15 (5), 257–259. 10.1016/s1056-8727(01)00155-6 11522500

[B96] Zarnegar A. Ong J. Matsyaraja T. Arora S. Chhablani J. (2023). Pathomechanisms in central serous chorioretinopathy: a recent update. Int. J. Retina Vitr. 9 (1), 3. 10.1186/s40942-023-00443-2 PMC9854068 36670451

[B97] Zhao Z. Zhang J. (2022). Nonhomogenous hyperreflectivity in the choriocapillaris layer on optical coherence tomography angiography implies early treatment with anti-VEGF for central serous chorioretinopathy. Ophthalmic Res. 65 (5), 506–515. 10.1159/000524488 35405684

